# Primary Hydatidosis of the Thigh Involving the Vastus Lateralis Muscle: A Case Report

**DOI:** 10.7759/cureus.56683

**Published:** 2024-03-22

**Authors:** Karthik Mohanan, Rajshree Dhadve, Karishma S Krishnani

**Affiliations:** 1 Radiodiagnosis, Dr. D. Y. Patil Medical College, Hospital and Research Centre, Dr. D. Y. Patil Vidyapeeth, Pune, IND

**Keywords:** daughter cysts, mri, soft tissue, intramuscular, hydatidosis

## Abstract

Hydatidosis is a parasitic disease caused by the larval stage of Echinococcal tapeworm that is endemic in many regions of the world. The subtle and nonspecific nature of the clinical signs frequently results in a delay in diagnosis. Ultrasonography (USG) is the first modality of choice for the diagnosis followed by magnetic resonance imaging (MRI). The detection of a ruptured membrane, observed as low-signal intensity on all pulse sequences, strongly suggests the diagnosis.

We report a rare case of a 39-year-old male who presented with fever, pain, and swelling in the right thigh. On further investigations, he was diagnosed to have primary hydatidosis of the thigh involving the vastus lateralis muscle and subcutaneous tissue. The patient underwent en bloc surgical excision of the mass and histopathology confirmed the diagnosis of intramuscular hydatidosis.

Understanding the diverse imaging manifestations of primary intramuscular hydatidosis is imperative for accurate preoperative diagnosis, thereby averting potentially fatal outcomes. Timely intervention is paramount as it mitigates both localized and systemic complications that may arise due to cyst maturation. This underscores the criticality of early therapeutic measures to enhance patient outcomes and diminish associated morbidities.

## Introduction

Human hydatid disease is a zoonotic parasitic infestation caused by echinococci [1]. The organism harbors in dogs and sheep, causing a health risk to humans [1]. Recently, hydatidosis has been observed even in developed countries, attributed to the rise in travel and tourism, extending its reach beyond traditionally endemic regions [2]. In India, the common states affected by the disease are Andhra Pradesh and Tamil Nadu [1].

The most commonly affected organ is the liver, followed by the lungs, brain, and other organs [3]. The musculoskeletal system is rarely affected by the disease, with the spine being the most common site, while muscles are affected in less than 1% of cases [4]. Soft tissue hydatid disease typically lacks symptoms and progresses slowly, often resulting in delayed diagnosis [4].

Recognizing the disease's epidemiological context provides crucial initial clues, while imaging modalities like ultrasonography (USG) and magnetic resonance imaging (MRI) serve as instrumental tools for confirmation [2]. USG is noninvasive, cost-effective, and readily accessible, making it a valuable first-line imaging modality for suspected cases [3]. MRI provides superior soft tissue resolution and detailed anatomical visualization, thereby helping in surgical planning and optimizing therapeutic strategies. MRI also helps differentiate hydatid cysts from other muscular pathologies, reducing diagnostic ambiguity [1]. The purpose of this study is to make future radiologists aware of how a timely definitive and accurate diagnosis can be done that guides subsequent therapeutic interventions.

## Case presentation

**Figure 1:**
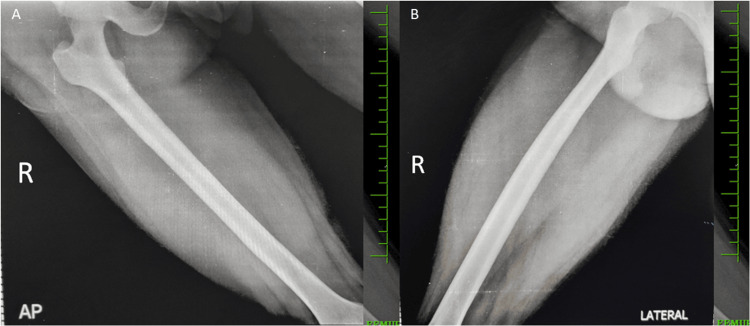
Radiograph of the right thigh: (A) anteroposterior and (B) lateral projection revealed no obvious abnormality.

A 39-year-old male presented with swelling of size approximately 10 cm x 5 cm in the anterolateral aspect of his right thigh for one month. The swelling was insidious in onset but was associated with pain and fever for the last one week. The patient was residing in a rural area, running a small grocery store, and was in close contact with a pet dog. No past surgical history or any family history was present. On physical examination, mild tenderness and local rise of temperature were noted. No other complaints like skin discoloration and discharge from the swelling were seen. Laboratory parameters were unremarkable. A plain radiograph of the right thigh did not reveal any significant findings (Figures 1A-1B).

**Figure 2:**
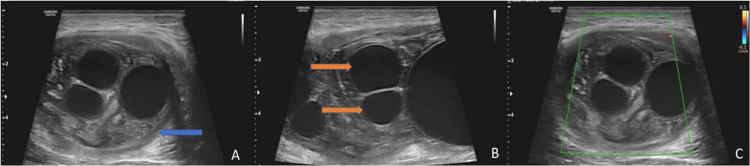
Ultrasonography (USG) images of the right thigh showing hydatid cyst. (A) Axial and (B) longitudinal sections revealing a large mother cyst(blue arrow) with a heterogeneous matrix and multiple smaller daughter cysts within (orange arrows). (C) The axial section of color Doppler USG revealing no significant vascularity within the lesion.

USG of the right thigh region showed a large heterogeneous solid-cystic mass in the vastus lateralis muscle, with multiple well-defined cystic anechoic areas measuring 13.5 cm x 6.7 cm x 5.1 cm (CC x AP x TR). The lesion showed no significant vascularity on the color Doppler (Figures 2A-2C).

Chest X-ray and abdominal ultrasonography revealed no abnormality.

**Figure 3:**
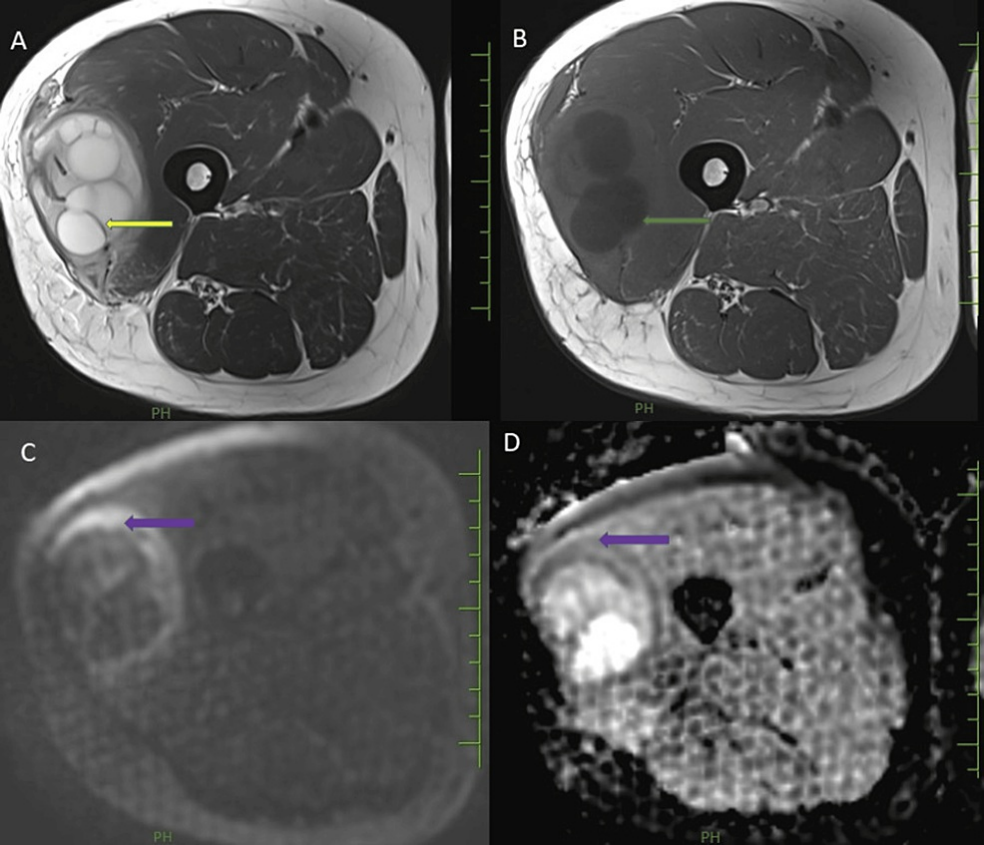
Axial MRI scans of the right thigh showing hydatic cysts. Multiple T2 hyperintense daughter cysts (A, yellow arrow), exhibiting hypointense signal intensity on T1-weighted images (B, green arrow). Diffusion restriction with a corresponding low ADC value was noted in the wall of the cyst (C and D, purple arrow). MRI, magnetic resonance imaging; ADC, apparent diffusion coefficient

3T MRI of the right thigh was done, which showed a large cystic lesion with multiple internal small daughter cysts measuring 13.5 cm x 6.7 cm x 5.1 cm (CC x AP x TR) involving the vastus lateralis muscle of the lateral compartment of the middle third of the right thigh and causing compression of vastus intermedius. The lesion appeared hyperintense on T2WI and hypointense on T1WI (Figures 3A-3B). There was no diffusion restriction noted within these cysts. However, peripheral diffusion restriction was noted in the wall with a corresponding low apparent diffusion coefficient (ADC) value.

**Figure 4:**
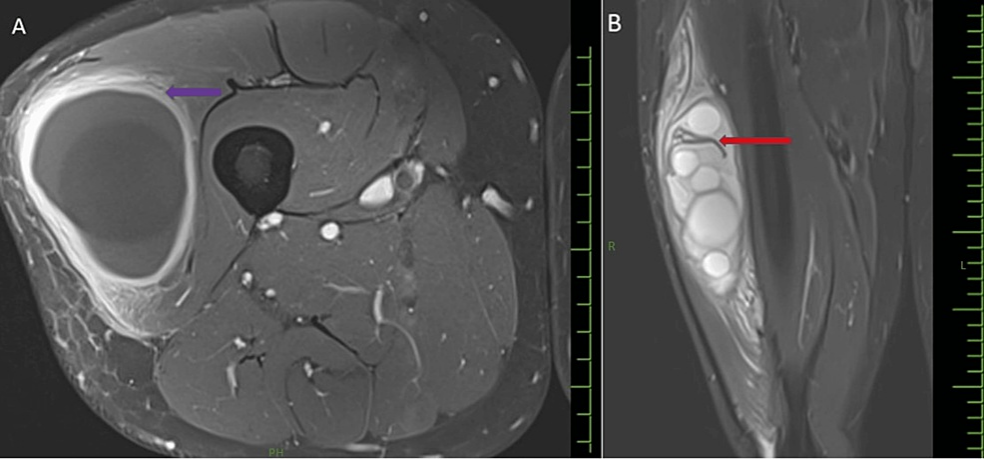
Gadolinium-enhanced MRI of the thigh: (A) axial showing the peripheral rim of enhancement (purple arrow); T2-weighted STIR coronal image (B) showing hypointense serpiginous foci within the cyst, consistent with the ruptured membrane (red arrow). MRI, magnetic resonance imaging; R, right; L, left; PH, posterior; STIR, Short Tau Inversion Recovery

In the post-contrast study, a peripheral rim of contrast enhancement was noted. Serpiginous foci within the mass, which are low signals on all sequences, consistent with the ruptured membrane (Figures 4A-4B).

They are specifically seen in hydatid disease.

**Figure 5:**
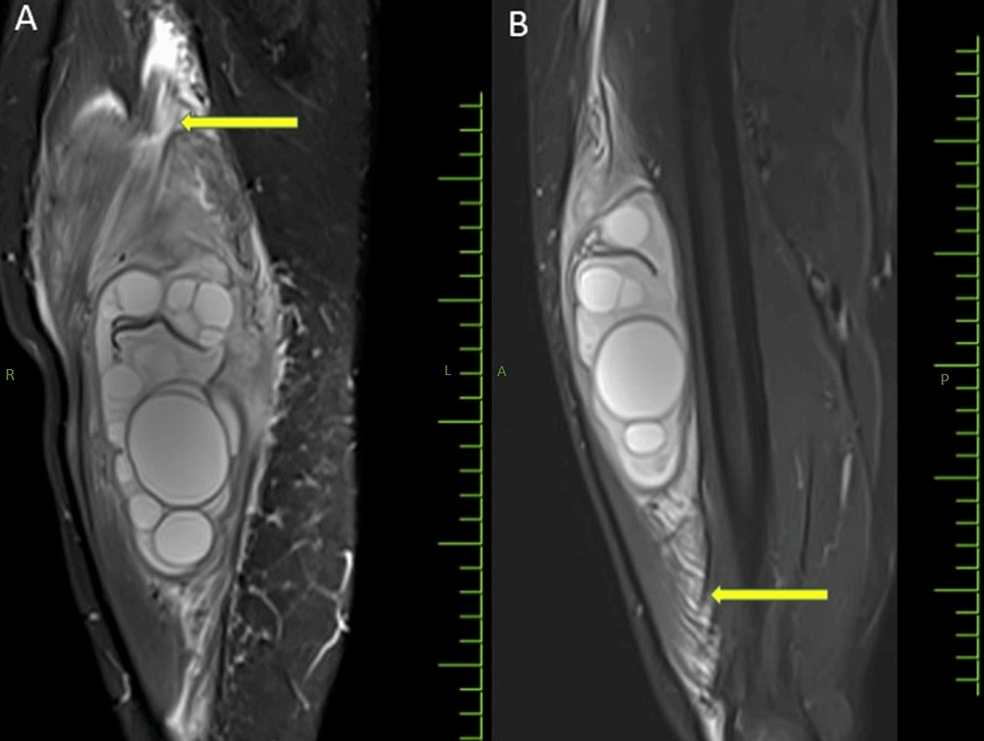
T2-weighted STIR MRI of the thigh: (A) coronal and (B) sagittal images showing ill-defined hyperintensities in the surrounding muscles adjacent to the hydatid cyst suggestive of inflammatory changes (yellow arrows). MRI, magnetic resonance imaging; STIR, Short Tau Inversion Recovery

Patchy T2WI/Short Tau Inversion Recovery (STIR) hyperintensities were noted in the muscles adjacent to the lesion, suggesting surrounding inflammatory changes (Figures 5A-5B).

Mild subcutaneous edema was also seen overlying the lesion.

**Figure 6:**
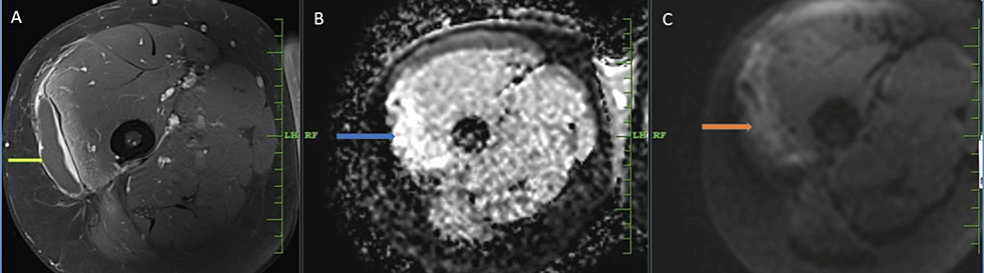
MRI of the right thigh showing collection. (A) Post-contrast T1-weighted axial image showing peripherally enhancing collection(yellow arrow) adjacent to the iliotibial band. (B) ADC image and (C) diffusion image, no diffusion restriction was noted within the collection (blue arrow in B, orange arrow in C). MRI, magnetic resonance imaging; ADC, apparent diffusion coefficient

A thin-walled, peripherally enhancing collection was observed beneath the iliotibial band, measuring 6.9 cm x 1.25 cm (AP x TR). It appeared hypointense on T1WI, mildly hyperintense on T2WI and STIR, with no diffusion restriction on the DWI sequence (Figure 6).

The patient underwent a CT brain, chest X-ray, and abdominal and pelvic sonography, which did not reveal any signs of hydatid disease. So, the radiological diagnosis of primary hydatidosis of the vastus lateralis muscle was accompanied by a probable secondary infection. No alternative differential diagnoses were identified based on the imaging studies conducted.

**Figure 7:**
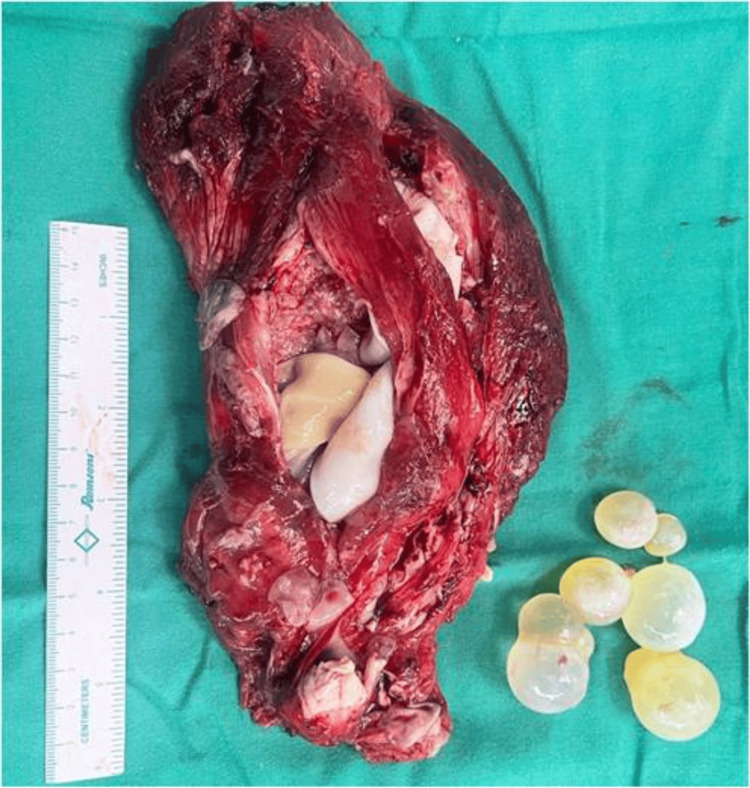
Postoperative excision specimen showing en bloc removal of the lesion with multiple daughter cysts.

The patient underwent en bloc excision of the cystic mass, preserving the cyst membrane with no complications during the surgery or in the immediate postoperative period (Figure 7).

Histopathological examination confirmed our imaging diagnosis of a hydatid cyst involving muscle.

Follow-up

On discharge, the patient was advised to continue albendazole 400 mg/BID treatment for one month. Follow-up was advised with USG of the thigh after 15 days, at one month, and three months. During the follow-up, the patient showed no clinical signs of recurrence. USG was repeated for three months. At the end of the three months, USG showed no features of residual cysts or recurrence.

## Discussion

This case showcases a rare occurrence of isolated primary intramuscular hydatid disease, potentially accompanied by secondary infection, in the thigh. The swelling went unnoticed until the patient experienced pain, likely indicative of infection. This emphasizes the significance of promptly addressing new symptoms in longstanding swellings. Advanced imaging is extremely useful for a complete evaluation of primary lesions as well as assessing associated complications in a single assessment, proving to be beneficial.

Hydatidosis is a parasitic disease caused by the larval stage of Echinococcal tapeworm that is contracted by ingestion of food or water contaminated with eggs of the parasite [5]. Echinococcus has two species, namely Echinococcus granulosus and Echinococcus multilocularis, causing cystic echinococcosis and alveolar echinococcosis in humans [3,5]. The natural habitat of Echinococcus is the small intestine of carnivores [5,6]. Africa, the Middle East, South America, the Mediterranean region, and a few others are known to be regions endemic to hydatidosis [7]. 

The liver is the most affected organ in humans (75% of cases), followed by the lung (15% of cases) [1]. Hydatidosis involving the musculoskeletal system is rare and accounts for less than 3% of all cases [8].

Hydatidosis of the musculoskeletal system can be secondary or primary [9]. In secondary hydatidosis, when there is hydatid disease involving the liver or lungs (primary locations), the parasites could cross the hepatic and pulmonary barriers to involve the muscles [9].

Primary hydatidosis involving the muscles is quite uncommon since high lactic acid levels and contractility of muscle tissue prove to be an unsuitable environment for the parasite to survive [1]. The proposed theory for primary hydatidosis is that after leaving the intestine, the embryo bypasses the liver and lungs, entering the systemic circulation where these organs act as filters [2].

Humans are not part of the natural life cycle of echinococcosis. Accidental ingestion of water contaminated with eggs of echinococcus species results in echinococcosis [5]. After the consumption of eggs, a larval stage known as the metacestode produces protoscoleces [5]. Though the exact period for the development of protoscoleces in human hosts is not known, it is proposed to be 10 months after ingestion of eggs [5].

Clinical symptoms tend to be nonspecific and insidious, often leading to a delay in diagnosis [10]. The nonspecific clinical picture could include a painless swelling that can progressively increase in size without any inflammatory features [7,11].

The initial period after the infection is mostly asymptomatic [5]. Patients may not develop any symptoms for years in cases of small cysts or cysts that are calcified and well-encapsulated. The presence of symptoms depends upon the size and number of the cysts, affected organs, pressure effects on surrounding structure, defense mechanism of the individual, and associated complications. Lung cysts are more likely to be symptomatic than hepatic cysts [5].

Immunodiagnostics tests such as enzyme-linked immunosorbent assay(ELISA), immunoelectrophoresis (IEP), indirect immunofluorescence test, and immunoblotting (IB) are commonly used laboratory tests for detection of serum antibodies [5]. These tests play a complementary role in primary diagnosis as well as in follow-up [4]. If purified antigens are used in both ELISA and IB, the specificity reaches up to 100%, with sensitivity values of 89% and 92%, respectively [4]. However, 10% to 20% of hepatic cysts and 40% of pulmonary cysts give false-negative results [5]. Less than 30% of patients show positive results in musculoskeletal hydatid disease [10]. Therefore, in practice, two different tests need to be used to get reliable results [5]. In our case, no serological tests were performed.

The initial radiological modality for detecting soft tissue hydatid disease should be USG, with sensitivity ranging from 95% to 100% in typical cases [10]. It can reveal the fluid nature of the soft tissue swelling, and in typical cases, a heterogeneous liquid formation with multiple daughter cysts can be visualized, as in our case [8,9]. CT is useful in enumerating the cysts, demonstrating size and topography, and detecting calcification of the cyst wall [12].

MRI is the best diagnostic tool to evaluate soft tissue hydatidosis, aiding in a thorough analysis of the cyst walls, internal structure, and locoregional extent with its superior contrast resolution [1]. On MRI, they usually show a cyst, with the characteristic appearance of multiple vesicles in a mother cyst, also known as *cysts or cysts within a cyst*. When compared to the mother cyst, these internal cysts (daughter cysts) are hypointense on T1-weighted imaging and hypo- or hyperintense on T2-weighted images [4]. The hydatid or endocyst membrane is shown by the pericystic hypointense line, which is more visible on T2-weighted imaging, as well as a second collagen-rich membrane known as the pericyst that results from the host's response to the parasite infection. A linear or ribbon-like hypointense signal inside the cyst, which corresponds to the *serpent sign*, indicates proliferous membrane detachment [11]. It depicts the hydatid cyst's involution as well as the detached and collapsing parasite membrane. Because of the pericyst's vascularization, the cysts may exhibit mild peripheral enhancement after gadolinium injection [1]. 

Complete surgical removal of the cyst is the best treatment so far in simple cases [5]. Hypertonic saline solution wash after cyst removal is mandatory to prevent protoscoleces dissemination. In situations where surgery is not possible, puncture-aspiration-injection-reaspiration (PAIR) or chemotherapy can be considered as alternative options [10]. The use of pre- and postoperative treatment with albendazole is still not clear. There is no consensus supporting its use in musculoskeletal hydatid disease [5,10].

## Conclusions

The present case emphasizes the need for considering intramuscular hydatidosis in slow-growing masses of extremities. Further evaluation with advanced imaging should be liberally considered for better evaluation and to avoid complications. If a patient presents with a new complaint of a long-standing swelling, in such cases, it is imperative to determine the underlying cause to rule out infection or rupture.

Our patient presented with swelling over the thigh. Detailed clinical history and multimodality imaging using USG and MRI of the thigh confirmed the diagnosis of intramuscular hydatid disease. The patient underwent surgical excision of the cyst along with albendazole therapy.

Thus, USG is a useful initial modality for diagnosis followed by MRI, providing superior analysis of the cyst structure and associated complications. It is imperative to make a preoperative diagnosis to prevent the eventuality of potentially life-threatening consequences. So when a patient comes from an endemic area, a hydatic cyst should always come in the differential diagnosis when a cystic lesion at a rare site is seen with typical imaging features. 

A multidisciplinary approach involving radiologists, surgeons, and infectious disease specialists is often necessary for optimal patient care. The differential diagnosis of hydatid cysts should always be considered when a patient from an endemic area presents with musculoskeletal swelling and reports a history of close contact with dogs, sheep, or cattle.
